# Fabrication of ethosuximide loaded alginate/polyethylene oxide scaffolds for epilepsy research using 3D-printing method

**DOI:** 10.3389/fbioe.2023.1244323

**Published:** 2023-12-01

**Authors:** Hatice Karabulut, Abir Dutta, Yunis Moukbil, Aysim Cisen Akyol, Songul Ulag, Banu Aydin, Rezzan Gulhan, Zeynep Us, Deepak M. Kalaskar, Oguzhan Gunduz

**Affiliations:** ^1^ Department of Systems Science and Industrial Engineering, State University of New York at Binghamton, Binghamton, NY, United States; ^2^ Center for Nanotechnology and Biomaterials Application and Research, Marmara University, Istanbul, Türkiye; ^3^ UCL Division of Surgery and Interventional Sciences, Royal Free Hospital Campus, London, United Kingdom; ^4^ School of Medicine and Psychology, College of Health and Medicine, Australian National University, Canberra, ACT, Australia; ^5^ Department of Bioengineering, Graduate School of Natural and Applied Sciences, Yildiz Technical University, Istanbul, Türkiye; ^6^ Department of Metallurgy and Materials Engineering, Faculty of Technology, Marmara University, Istanbul, Türkiye; ^7^ Department of Biophysics, School of Medicine, Marmara University, Istanbul, Türkiye; ^8^ Department of Medical Pharmacology, School of Medicine, Marmara University, Istanbul, Türkiye; ^9^ Epilepsy Research and Implementation Center, Marmara University, Istanbul, Türkiye

**Keywords:** 3D-printing, drug resistant epilepsy, epilepsy treatment, polyethylene oxide, implantable scaffolds, sodium alginate

## Abstract

Epilepsy is a medical condition that causes seizures and impairs the mental and physical activities of patients. Unfortunately, over one-third of patients do not receive adequate relief from oral Antiepileptic Drugs (AEDs) and continue to experience seizures. In addition to that, long term usage of Antiepileptic Drugs can cause a range of side effects. To overcome this problem, the precision of 3D printing technology is combined with the controlled release capabilities of biodegradable polymers, allowing for tailored and localized AED delivery to specific seizure sites. As a result of this novel technique, therapeutic outcomes can be enhanced, side effects of AEDs are minimized, and patient-specific dosage forms can be created. This study focused on the use of ethosuximide, an antiepileptic drug, at different concentrations (10, 13, and 15 mg) loaded into 3D-printed sodium alginate and polyethylene oxide scaffolds. The scaffolds contained varying concentrations (0.25%, 0.50%, and 0.75% w/v) and had varying pores created by 3D patterning sizes from 159.86 ± 19.9 µm to 240.29 ± 10.7 µm to optimize the releasing system for an intracranial administration. The addition of PEO changed the T_g_ and T_m_ temperatures from 65°C to 69°C and from 262°C to 267°C, respectively. Cytotoxicity assays using the human neuroblastoma cell line (SH-SY5Y) showed that cell metabolic activity reached 130% after 168 h, allowing the cells to develop into mature neural cells. *In vitro* testing demonstrated sustained ethosuximide release lasting 2 hours despite crosslinking with 3% CaCl_2_. The workpaves the way for the use of ethosuximide -loaded scaffolds for treating epilepsy.

## 1 Introduction

Epilepsy is a serious neurological disease which is characterized by frequent, repeated seizures. It is a disabling condition that can have severe effects on the patient’s life including societal consequences (Swarztrauber et al., 2003; Beghi, 2020). Diagnosis and treatment of the disease pose many difficulties for both the patient and clinicians. Presently, 70 million individuals worldwide suffer from epilepsy (Kalilani et al., 2018). Most of the patients are treated with oral antiepileptic drugs (AED). However more than one-third of people with epilepsy who take more than two AED still experience seizures, and epilepsy is known to be highly resistant to pharmaceutical treatment (Shorvon, 1996; Sander, 2003). Alternative approaches to treating epilepsy are therefore required.

The presence of higher levels of drug in body than the brain leads to systemic side effects. The blood vessels in the brain have many properties that prevent substances from passing through the blood flow to the brain (Halliday et al., 2012; Bonilla et al., 2021). These properties could inhibit the transport of therapeutic agents to the brain. For this reason, some drugs need to be used in higher doses. Often, these high serum levels are responsible for peripheral side effects and interruptions of drug therapy. As opposed to targeting the seizure focus specifically, AEDs affect all neurons in the brain. As a result, cerebral side effects such as ataxia, sedation, diplopia, and mood swings may occur (Halliday et al., 2012).

‘Seizure focus’ refers to the point in the brain where abnormal seizures begin and develop. This reserved area of the brain is the therapeutic target of the anti-epileptic drug release systems (Thijs et al., 2019). However, the fact that the drugs act by dispersing to the whole brain poses a problem in terms of creating side effects, as it is unnecessary to distribute throughout the entire brain and body. Localizing the seizure focus and applying AEDs to this area provides high concentrations of drug production in the seizure focus. This not only alleviates seizures but also eliminates many of the peripheral and central side effects that occur with the oral administration of high doses (Halliday et al., 2012). Therefore, focal drug administrations be alternative treatments for patients with refractory epilepsy (Chen et al., 2015). Practical and clinically viable methods of delivering AEDs directly to the seizure focus have emerged over the past decade and are evolving rapidly (Schmidt and Löscher, 2005). Intracranially implantable drug delivery systems with proven potential in the treatment of focal neurological disorders allow AEDs to be delivered directly to the seizure focus over extended periods of time (Montanheiro et al., 2022). Furthermore, based on the types of seizures: generalized seizures and focal seizures, different antiepileptic drugs are preferred in the treatment. Ethosuximide (ETHX), a water-soluble weak acid with a molecular weight of 141.17, is one of the old classical anticonvulsant drugs used in the treatment of the absence epileptic seizures (Wu et al., 2020).

Biodegradable polymers are gradually broken down into their monomers in the presence of water and enzymes. When these polymers are combined with drugs and implanted within the body, the polymer progressively dissolves and the drug it contains is released into the environment (Chen et al., 2015). This process continues until the polymer is completely degraded, eliminating the need to surgically remove the waste implant (Halliday et al., 2012). Although biological polymers have good biocompatibility, they are generally poor in mechanical properties. The necessity of maintaining the biological features of biopolymers makes them more difficult to work with and raises the cost of manufacture or recovery (Lorente et al., 2022). As a result, polymer blends which are physical mixtures of polymers or copolymers that interact with secondary forces are preferred in order to prepare mixtures of polymeric materials with the desired properties (Song et al., 2018).

Poly (ethylene oxide) (PEO) is a linear hydrophilic biodegradable polymer derived from ethylene oxide. It is simple to manufacture, water-soluble, non-toxic, and not affected by pH or physiological fluids (Çaykara et al., 2005). When the PEO is in contact with water, it becomes hydrated and swollen, forming a layer of hydrogel that controls the further entry of water and the dissolution of any active substances that may be present inside the polymer (Saquing et al., 2013). For the manufacture of drug release systems, the PEO’s unique hydration and swelling capabilities make it an attractive option. Alginates are anionic, hydrophilic polysaccharides found in brown seaweed and bacteria (Hegde et al., 2022). Because of intrinsic features such as biocompatibility, biodegradability, nontoxicity, high mechanical strength, abundance, and high adsorption capacity, alginate-based polymeric systems are a promising material in biomedical applications such as drug administration (Wu et al., 2005). Since the hydroxyl (-OH) groups on SA can interact with the ether oxygen in PEO via hydrogen bonds, PEO is suitable for SA blends (Zia, 2017; Mozafari et al., 2019). Furthermore, because both polymers are water soluble, the likelihood of allergic responses is reduced. Additionally, the hydrogen bond interaction with the increasing SA concentration enhanced the young modulus, stress, and elongation at break of PEO/SA (Zia, 2017). Therefore, a PEO blend with SA was chosen as a suitable candidate (Saquing et al., 2013).

To produce a 3D implantable scaffold there are various techniques that can be used such as 3D printing, electrospinning, and casting (Picco et al., 2022). Gel formulation, another technique for creating scaffolds, has limited shape and size options due to its high hydrophilicity. The hydrophilic nature of the gel may also result in poor mechanical strength and stability (Abasalizadeh et al., 2020). 3D printing is a method for creating complex three-dimensional structures with customized dimensions and shapes using a computer-aided deposition of polymeric scaffolds (Mohammed et al., 2021). It is a promising additive manufacturing process, may be used to create biostructures with appropriate biological, structural, and mechanical qualities (Mozafari et al., 2019). As a result, 3D printing allows for the development of complex geometries with homogenous cell distributions, offering the advantage of creating safe, stable, and cost-efficient implantable scaffolds (Daemi and Barikani, 2012). Therefore, the choice of 3D printing over gel formulation for scaffold drug delivery system depends on various factors such as the desired shape, size, porosity, mechanical strength, drug loading, release profile, biocompatibility, and cost-effectiveness of the scaffold (Kuo and Ma, 2001; Zhu and Marchant, 2011). Novel 3D-printing technology increases therapeutic efficacy and tolerance, giving patients a higher quality of life. This is especially important for patient’s suffering for chronic conditions, for instance schizophrenia or Parkinson’s disease (Picco et al., 2022). Additionally, 3D printing offers an effective clinical solution for the treatment of complex diseases, such as epilepsy, Alzheimer’s disease, and cancer (Calori et al., 2020). Besides there are multiple studies about 3D printed implantable scaffolds for previously mentioned diseases, the research is continuing to overcome current challenges and to develop an implantable scaffold for the treatment of epilepsy by using 3D-printing technology.

This study considers developing an implantable AED containing 3D printed scaffolds and also it aims to direct targeting of AED to the seizure focus. Targeting will be achieved by delivering AEDs directly to the brain parenchyma through an intracranial implant. Thanks to this intracranial administration, systemic toxicity can be decreased since AEDs can pass through brain blood barrier (BBB) (Pathan et al., 2010). Because of continuous release from a matrix, implants can achieve a longer duration of drug exposure. SA and PEO, biodegradable polymers, are used for constructing drug-releasing implants. 3D-printing technology was used to construct a scaffold with the desired shapes and mechanical properties at a lower cost. In this work, ethosuximide, an anti-epileptic drug, was chosen as a therapeutic agent to overcome absence epileptic seizures. The implants were characterized, and their drug release profiles were obtained.

## 2 Materials and methods

### 2.1 Materials

Sodium alginate, (Mw = 216,000 g/mol), poly-ethylene oxide (Mw = 300,000 g/mol) and ETHX (E−7138) was obtained from Sigma-Aldrich (Germany). Calcium chloride dihydrate (CaCl_2_·2H_2_O) was bought from Merck (Darmstadt, Germany) to use as crosslinking agent. Phosphate buffer saline (PBS) (pH 7.4) was purchased from Chembio (Istanbul, Turkey).

### 2.2. Design and fabrication of scaffolds

The design of the scaffolds was drawn using a three-dimensional Solidworks program as a square shape with dimensions of 20 mm × 20 mm based on the optimized scaffold structure in the previous study (Aranci et al., 2020). Simplify3D software was used to convert the programmed design of scaffolds into G-codes to enable printing with an extrusion 3D printer (Hyrel 3D, SDS-5 Extruder, GA, USA). A plastic 10 mL syringe filled with polymer solution was connected to the printer with a metallic needle tip of 30 gauge (0.160 mm). Process parameters were adjusted, such as 96% of infill density, 10 mm/s printing speed, 1 mL/h flow rate, and 8 layers.

### 2.3 Preparation of polymer solutions

Each polymer and anti-epileptic drug were weighed (Precisa XB 220A SCS, Germany) prior to dissolve in distilled water. Firstly, sodium alginate (SA) was dissolved at a concentration of 4.5% (weight/volume, w/v) (Aranci et al., 2020), and the solution was homogenized using a magnetic stirrer (Wise Stir^®^, MSH-20A, Wertheim, Germany) for an hour at 450 rpm. After obtaining clear and homogenous solutions of SA, 3 different concentrations of poly-ethylene oxide (PEO) were separately added at concentrations of 0.25%, 0.50%, and 0.75% (w/v) to obtain composite polymer solutions and mixed for an additional 1 h under the same stirring conditions. Finally, the antiepileptic drug ETHX was added to 10 mL of the 4.5% SA/0.75% PEO composite polymer solution whose absolute added drug weights were 10, 13, and 15 mg and mixed for another 30 min. The clinically dose of ETHX drug were decided based on previous studies and amount of polymer used for printing (Gülhan Aker et al., 2010).

### 2.4 Crosslinking procedure of printed scaffolds

The surface of each printed scaffold was sprayed with a crosslinking agent of CaCl_2_ solution at a concentration of 1%. Firstly, 1 g of calcium chloride dihydrate (CaCl_2_·2H_2_O) was dissolved in 100 mL of distilled water and placed in a standard spray bottle. The CaCl_2_ solution was sprayed on the surface of the scaffold at 45 angles at a distance of 10 cm. The same procedure was applied to both surfaces.

### 2.5 Characterization of printed scaffolds

Morphological analysis of scaffolds was performed using scanning electron microscopy (SEM, EVA MA 10, ZEISS, Pleasanton, CA, USA) after coating the scaffolds’ surfaces with gold. The average pore created by 3D patterning size and distribution of each scaffold were analysed with imaging software (Olympus AnalySIS, Waltham, MA, USA).

Fourier Transform Infrared (FT-IR) Spectrometer (Jasco, FT-IR 4700) was used to analyse the chemical characteristics of the scaffolds at a scanning range of 400 and 4000 cm^-1^ and a resolution of 4 cm^-1^.

Thermal behaviour of the scaffolds was analysed using differential scanning calorimetry (DSC, Shimadzu, Tokyo, Japan) at temperatures ranging from 25°C to 350°C with a scanning rate of 10°C/min.

A mechanical tensile test device was used to determine the scaffolds’ mechanical characteristics (SHIMADZU, EZ-LX, Beijing, China) after measuring each scaffold’s length and thickness using a digital micrometre (Mitutoyo MTI Corp., USA). Thickness, width, and length of each scaffold are shown in the Table 1, below. The three samples of each scaffold were tested from their upper and lower regions, and all samples were positioned uniaxially to be tested using the apparatus of the mechanical tensile test device.

**TABLE 1:** Dimensions of mechanical tensile testing samples.

Samples names	Thickness (mm)	Width (mm)	Length (mm)
4.5% SA	0.043	10	20
4.5% SA/0.25% PEO	0.024	10	20
4.5% SA/0.50% PEO	0.027	10	20
4.5% SA/0.75% PEO	0.032	10	20
4.5% SA/0.75% PEO/10 mg ETHX	0.070	10	20
4.5% SA/0.75% PEO/13 mg ETHX	0.069	10	20
4.5% SA/0.75% PEO/15 mg ETHX	0.086	10	20

In order to determine the viscosity of the solutions at room temperature, a digital viscometer (CP 2000 Plus, Lamy Rheology, Champagne au Mont d'Or, France) was used to measure the shear rates from 0 to 500 s^−1^. Aqueous solutions of each polymer solution were prepared by gentle stirring for 4 h and air bubbles were eliminated. Therefore, the solution was allowed to sit overnight.

The swelling test of the printed scaffolds was performed within different time intervals in phosphate-buffered saline (PBS) (pH 7.4) at 37°C to measure the capacity of the printed scaffolds performance in terms of water retention. Each sample was kept in a 0.5 mL PBS solution at 37°C in a thermal shaker incubator (BIOSAN TS-100). The weight of all printed scaffolds (W_0_) was measured, and wet sample weights (W_W_) were measured at different times. Furthermore, the swelling ratio (SR) of the printed scaffolds was calculated using the equation (Koyun et al., 2023):

$$SR=\frac{W_{W}-W_{0}}{W_{0}}\times 100$$

The degradation (weight loss) of the printed scaffolds was performed at different time intervals in 0.5 mL PBS solution (pH = 7.4), in the thermal shaker at 37°C. PBS medium was taken out each time interval and the swollen scaffolds were weighed and dried at 37°C for a day. The degradation was calculated by evaluating weight loss (%) using the equation (Ghosh Dastidar et al., 2023):

$$Degredation\;rate\;\left(\%\right)=\frac{W_{1}-W_{2}}{W_{1}}\times 100$$

Each printed scaffolds were divided into small pieces (10 mm × 10 mm), which were then submerged in 1 mL of phosphate buffer saline (PBS; pH:7.4) and the samples were then incubated at 37°C in a thermal shaker (BIOSAN TS-100) at 360 rpm. Various time intervals were used to measure the anti-epileptic drug ETHX‘s release profile: 15 min, 30 min, and 60 min, then 1-h intervals. A UV-1280 spectrophotometer (SHIMADZU, 190–300 nm) was used to measure the PBS after the time intervals. 1 mL of new PBS was added to the current scaffolds for the next measurements after each measurement. The concentrations of the discharged solution were determined using a graph and a standard calibration curve. The predetermined anti-epileptic ETHX drug concentrations (0.2, 0.4, 0.6, 0.8, and 1 μg/mL) were prepared and analysed using the UV-1280 spectrophotometer. The highest absorbance values from the calibration curve were used to generate the absorbance graph. Several mathematical models, such as zero-order (Eq. 1), first-order (Eq. 2), Higuchi (Eq. 3), Korsmeyer-Peppas (Eq. 4), and Hixson-Crowell (Eq. 5), were applied to calculate the ETHX drug kinetic release from the drug-loaded printed scaffolds. Their individual equations are provided below (Koyun et al., 2023):

$$Q=K_{0}t$$ (1)

$$\text{In}\left(1‐Q\right)=‐K_{1}t$$ (2)

$$Q=K_{h}t^{1/2}$$ (3)

$$Q=Kt^{n}$$ (4)

$$Q^{1/3}=K_{hc}t$$ (5)

The kinetic constants for each model are K, K_0_, K_1_, K_h_, and K_hc_. Q represents the fractional quantity of drugs released at time t in these equations. The diffusion exponent, abbreviated n, reflects the drug’s mechanism of release.

### 2.6 Cytotoxicity of printed scaffolds

To test the cytotoxicity of drug loaded scaffolds, SH-SY5Y cells (a human neuroblastoma cell line) were used. The initial cell seeding concentration was 10^5^ cell/well.

For human neuroblastoma cell line culture, SH-SY5Y (CRL-2266™, ATCC), were supplemented with 10% (v/v) inactivated Fetal Calf Serum (FCS) (Thermo Fisher Scientific), 2 mM L-glutamine (Sigma), 100 IU/mL penicillin and streptomycin in 25 cm^2^ flasks at the cell density of 10^6^ cells/mL in Dulbecco’s Modified Eagles Medium (DMEM, Gibco). They were then cultured in a humidified atmosphere of 5% CO_2_ at 37°C. The medium was replaced every 2 days and the cell cultures were split twice a week. Cells at about 80% of confluence were trypsinized (0.05%, trypsin-EDTA, Gibco), washed and scored for viability. Culture medium was replaced with fresh one after overnight recovery.

Seeded SH-SY5Y cells were treated with various concentrations of drug-loaded scaffolds (ETHX10, 13, 15 mg) and the plates were incubated in at 37°C in a 5% CO_2_ incubator for 24, 48, 72, and 168 h. After the incubation period cells were incubated for 3 h with 5 mg/mL of MTT (Sigma), dissolved in phosphate buffered saline (PBS). Followed by the addition of DMSO (200 μL) and then gentle shaking for 2 min so that complete dissolution. Absorbance was recorded at 550 nm using the microplate spectrophotometer system (Synergy H1 Hybrid Multi-Mode Reader). All MTT assays were repeated three times. Morphological investigations were performed after each treatment.

For DAPI staining cells grown as described above were exposed to ETHX for the indicated times. After treatment, the cells were washed with phosphate-buffered saline (PBS) and then fixed in ice-cold acetone for 30 min. The cells were then washed twice with PBS and stained with DAPI (300 nM) for 10 min in the dark. Images were captured using a Leica DMI 6000B fluorescence microscope (Wetzlar, Germany).

The statistical analysis was carried out with GraphPad Prism 9 (San Diego, CA, USA). Data were analysed using the one-way ANOVA test. The level of significance was set to *p < 0.05, **p < 0.01, and ***p < 0.001.

## 3 Results and discussion

**FIGURE 1:**
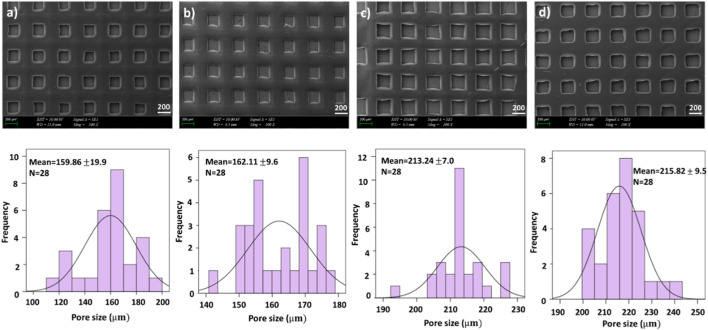
SEM images of printed scaffolds **(A)** 4.5% SA, **(B)** 4.5% SA/0.25% PEO, **(C)** 4.5% SA/0.50% PEO, **(D)**4.5% SA/0.75% PEO and the corresponding graphical representations of pore created by 3D patterning size distributions.

**FIGURE 2:**
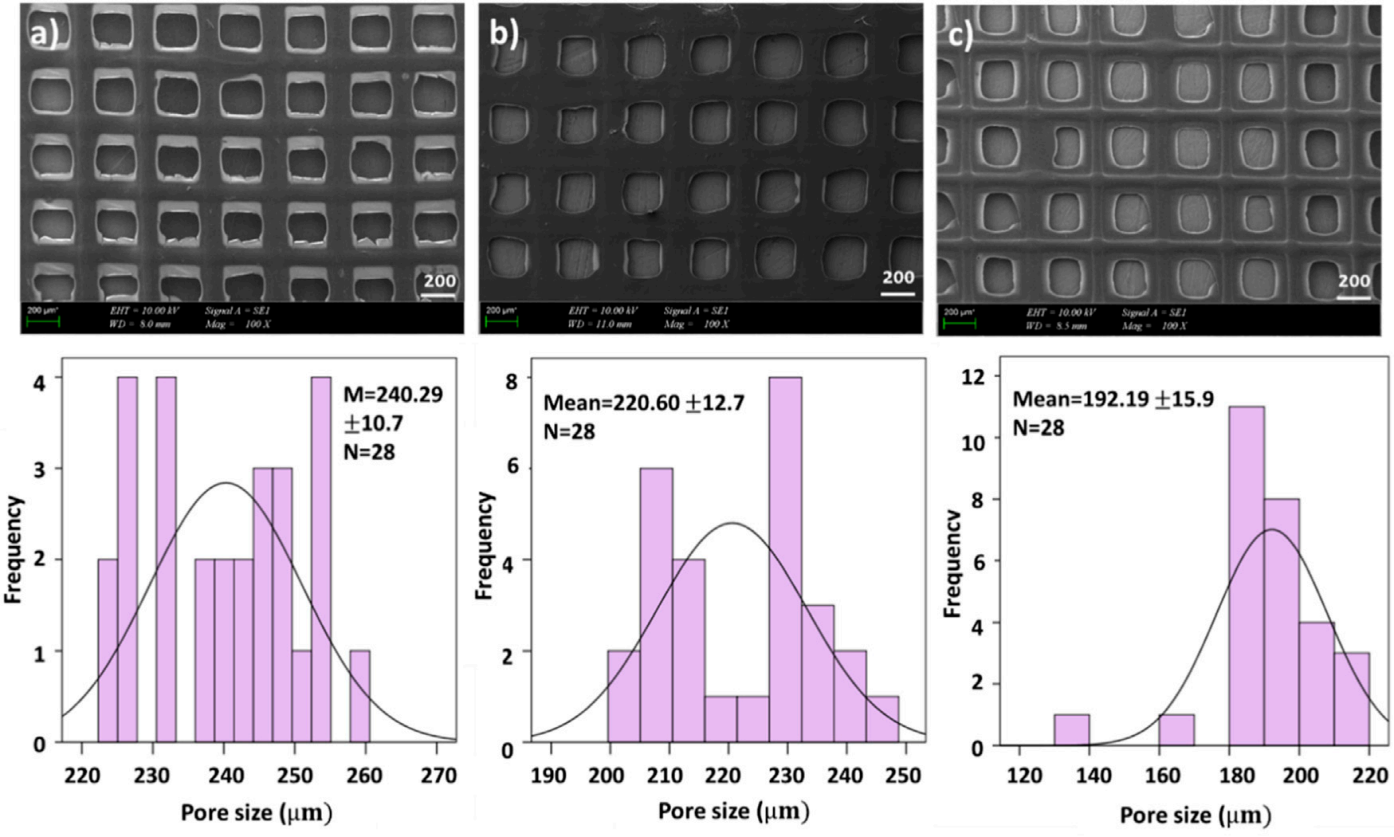
SEM images of drug loaded printed scaffolds with **(A)** 4.5% SA/0.75% PEO/10 mg ETHX, **(B)** 4.5% SA/0.75% PEO/13 mg ETHX, **(C)** 4.5% SA/0.75% PEO/15 mg ETHX and the corresponding graphical representations of pore created by 3D patterning size distributions.

The first test we conducted was examining the morphology of the scaffolds using scanning electron microscopy (SEM), which allowed us to clearly see the pore created by 3D patterning size distributions of each scaffold as is shown in Figure 1 and Figure 2. Figure 1 and Figure 2 exhibited that adding PEO and ETHX to the scaffold changed the morphological structure of the scaffolds and pores created by 3D patterning of all scaffolds were clearly visible without any clogging issues. Each scaffold displayed a similar open pattern structure. Although there was not much notable difference in the pattern size distribution of the scaffold, the mean pore created by 3D patterning size value of the pure alginate-based scaffold was 159.86 ± 19.9 µm which was lower than the PEO-added scaffold. When the PEO concentration was 0.25% in the scaffold, it resulted in the lowest pore created by 3D patterning size value, which was 162.11 ± 9.6 µm but when the PEO concentration was highest, the pore created by 3D patterning size increased to 215.82 ± 9.5 µm.

In Figure 2, the effects of adding ETHX to the scaffolds can be seen. When the drug concentration was the lowest, it showed the highest pore created by 3D patterning size among all scaffolds which measured at 240.29 ± 10.7 µm. Besides that, the pore created by 3D patterning size of the drug-loaded scaffold gradually decreased with the increase in drug concentration. Nonetheless, the pore created by 3D patterning size of the scaffold with the highest drug load was lower than that of the control group (4.5% SA/0.75% PEO) whereas 4.5% SA/0.75% PEO/15 mg ETHX had 192.19 ± 15.9 µm pore created by 3D patterning size.

**FIGURE 3:**
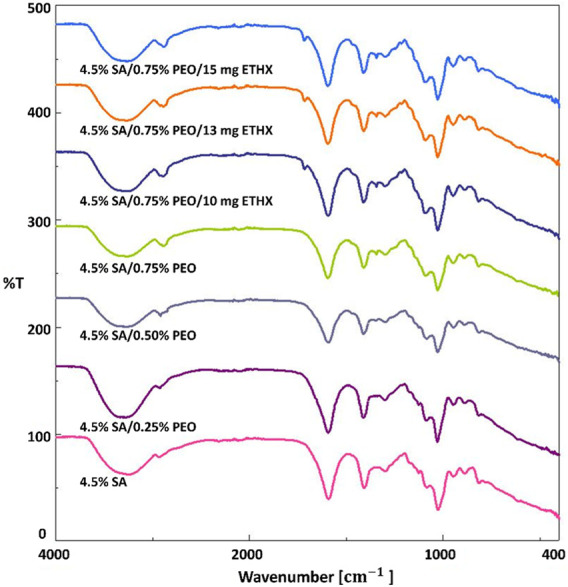
FTIR spectra of printed scaffolds.

Some unique functional groups like hydroxyl, ether, and carboxylic groups are associated with absorption bands of sodium alginate-based scaffolds (Daemi and Barikani, 2012). Therefore, three main regions were observed and focused on in regard to the absorption bands of sodium alginate-based scaffolds, which are shown in Figure 3, are the band in the range of 3700–2600 cm^-1^, in the range of 1700–1540 cm^-1^ and in the range of 1115–973 cm^-1^. The spectra band in the range of 3700–2600 cm^-1^ was observed due to stretching vibrations of the O–H bond and aliphatic C–H bond stretching vibrations of the sodium alginate-based scaffolds (Hou and Wu, 2019; Han et al., 2010). The spectra band between 1700 cm^-1^ and 1540 cm^-1^ was related to the vibration of the asymmetric and symmetric stretching of the C–O–O bond. The spectra band in the range of 1115–973 cm^-1^ happened due to C–O stretching vibration, C–C–H and C–O–H deformation (Hou and Wu, 2019; Pongjan and yakul, 2009). According to the literature (Hou and Wu, 2019), the spectra band at 3234 cm^-1^ was observed and it shows the characteristic stretching vibrations of the O–H bond of sodium alginate. Furthermore, the vibration of the symmetric stretching of the C–O–O bond at 1406 cm^-1^ and the vibration of the C–O–C stretching vibration peaks at 1082 cm^-1^ were observed (Zheng et al., 2016; Mimmo et al., 2005). Changes in the FTIR absorption spectra band of some peaks of the components in a blended system are considered evidence of energetic interactions, consequently, depending on the strength of the intermolecular connections. The incorporation of PEO into sodium alginate-based scaffolds caused some changes. Because of CH_2_ stretching, C–H bending, and wagging vibrations of CH_2_, the characteristic spectra bands of PEO added scaffolds resulted as 2888 cm^-1^, 1592 cm^-1^ and 1342 cm^-1^ (Puci and Jurkin, 2012). The spectra at 3234 cm^-1^ were shifted to 3290 cm^-1^ with the addition of PEO. Moreover, the addition of ETHX resulted in a particular spectra band at 1714 cm^-1^ which was related to C–O stretching vibration (Vijaya Chamundeeswa et al., 2011). The addition of PEO and ETHX resulted in some shifts in the spectra bands, which means that encapsulation of ETHX drug in the printed scaffolds was successfully achieved using the 3D printing method for the fabrication of the printed scaffolds.

**FIGURE 4:**
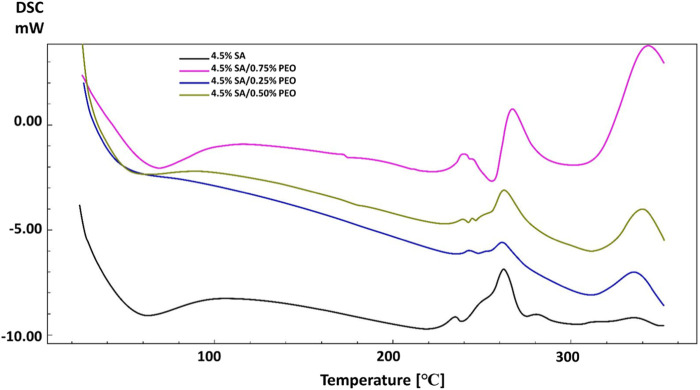
DSC analysis of scaffolds in the absence of anti-epileptic ethosuximide drug.

DSC analysis was performed to analyse the thermal characteristics of the scaffolds. As can be seen in Figure 4, pure SA and the addition of PEO showed different thermal points. The glass transition temperature (T_g_) and melting temperature (T_m_) of pure SA were observed at 65°C and 262 °C (Siddaramaiah et al., 2008). Although PEO has lower T_m_ (69 °C), the addition of PEO improved the thermal characteristics of the pure SA and the high concentration of PEO in the printed scaffolds resulted in higher glass transition temperatures (T_g_) and melting temperatures (T_m_) (Ibrahim and Johan, 2012). These T_g_ and T_m_ temperatures changed from 65°C to 69°C and 262°C–267 °C respectively, when the PEO concentration was highest in the scaffold. This result proves the energetic interactions and intermolecular connections between pure SA and PEO according to changes in the melting temperature (Ozturk et al., 2022; Karabulut et al., 2023).

**FIGURE 5:**
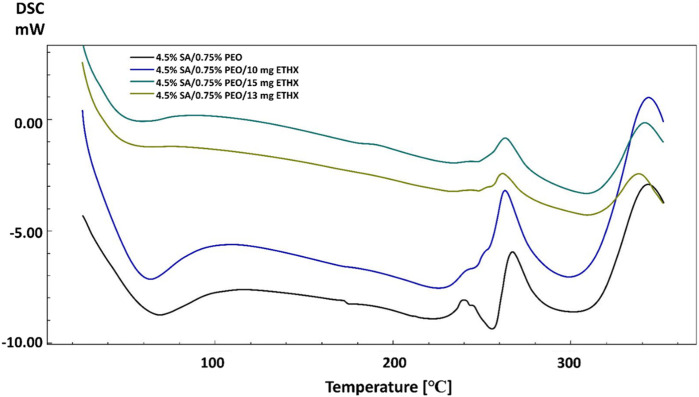
DSC analysis of scaffolds including ETHX.

The addition of the anti-epileptic ETHX drug also caused some changes in the thermal properties of the scaffolds. When the results of scaffolds including ETHX are compared to the control group (4.5% SA/0.75% PEO), a slight decrease was observed in the T_g_ of the control group with the increase in the amount of ETHX (Figure 5). The T_g_ temperature of the pure SA printed scaffold decreased to 58 °C when the ETHX concentration was the highest in the control group. However, a small increase was observed in the temperature of T_m_ of control group when the ETHX concentration was highest and analysed at 263 °C.

The mechanical properties of the scaffolds were tested using a tensile test, and the results are shown in Table 2. The mechanical characteristics of the scaffolds were altered with the addition of PEO and ETHX, as well as a change in the concentration of these two materials. Additionally, the porous structure of the scaffolds could affect their mechanical properties (Stangl et al., 2001). When the results of each sample were compared, we can say that the highest tensile strength value was observed on the samples of pure alginate-based scaffold. The addition of PEO decreased the tensile strength and elongation at break, and this might be due to weak interaction within the components and the presence of the crosslinking agent CaCl_2_ (Moon et al., 2009). However, it was obvious that the increase in the concentration of PEO resulted in a gradual and slight increase in the tensile strength and elongation at break value compared to the pure alginate-based scaffold. The tensile strength value of 4.5% SA/0.25% PEO scaffold was 4.04 ± 3.72 MPa, but a higher tensile strength value was obtained in 4.5% SA/0.75% PEO scaffold, which was 6.21 ± 2.31 MPa. Furthermore, the elongation at break value of the 4.5% SA/0.75% PEO scaffold (2.44 ± 0.10) was higher than that of the pure alginate-based scaffold (2.23 ± 1.34 MPa). On the other hand, when the ETHX concentration was increased, the mechanical properties of the 4.5% SA/0.75% PEO decreased. The highest mechanical test of a drug loaded scaffold was measured when the drug concentration was 10 mg, and the tensile strength reached 3.58 ± 2.19 MPa. The Young’s modulus was obtained from the slope of the strain-strain graph, keeping the strain value constant at 0.2% and results indicated that the 4.5% SA had the maximum Young modulus value (28.75 ± 1.76 MPa) and the minimum Young modulus (3.5 ± 0.56 MPs) belonged to the 4.5% SA/0.75% PEO/15 mg ETHX scaffold.

**TABLE 2:** Mechanical testing results of the scaffolds.

Samples names	Tensile strength (MPa)	Elongation at break (%)	Young modulus (MPa)
4.5% SA	6.42 ± 5.54	2.23 ± 1.34	28.75 ± 1.76
4.5% SA/0.25% PEO	4.04 ± 3.72	0.98 ± 0.62	14.5 ± 0.42
4.5% SA/0.50% PEO	5.06 ± 2.51	2.07 ± 1.43	23.5 ± 2.82
4.5% SA/0.75% PEO	6.21 ± 2.31	2.44 ± 0.10	26.45 ± 1.06
4.5% SA/0.75% PEO/10 mg ETHX	3.58 ± 2.19	2.63 ± 0.64	15.2 ± 2.82
4.5% SA/0.75% PEO/13 mg ETHX	1.85 ± 0.61	2.34 ± 0.77	7.5 ± 0.72
4.5% SA/0.75% PEO/15 mg ETHX	1.04 ± 1.04	1.74 ± 0.59	3.5 ± 0.56

**FIGURE 6:**
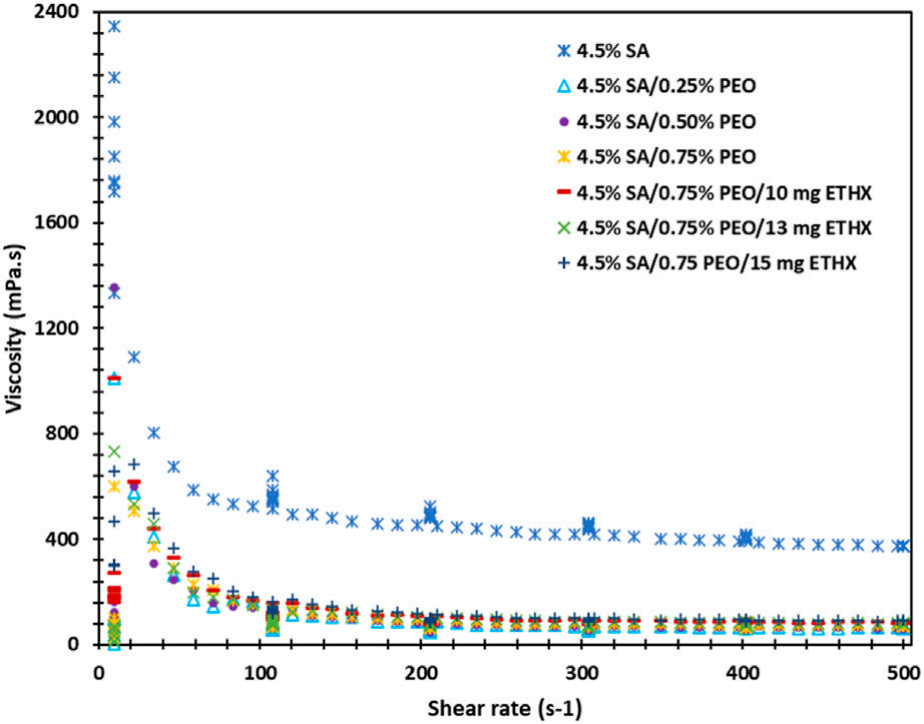
The graph of the viscosity values of each solution per time.

The rheology analysis of each solution was performed, and results can be seen in the Figure 6. Each solution resulted in a different behaviour of viscosity, and this might be due to several reasons, such as the presence of macro-ions and counter-ions, primarily the molecular weight, the chain rigidity, and the solvent quality (Belalia and Djelali, 2014). The highest viscosity was resulted in the control group of pure alginate solution. However, with the addition of PEO polymer or ETHX drug, the viscosity of the solutions pattern was fluctuated which might be due to the presence of intermolecular junctions (Belalia and Djelali, 2014). Additionally, the shear rate and viscosity showed an inverse correlation, and the result can be seen in the supplementary file.

**FIGURE 7:**
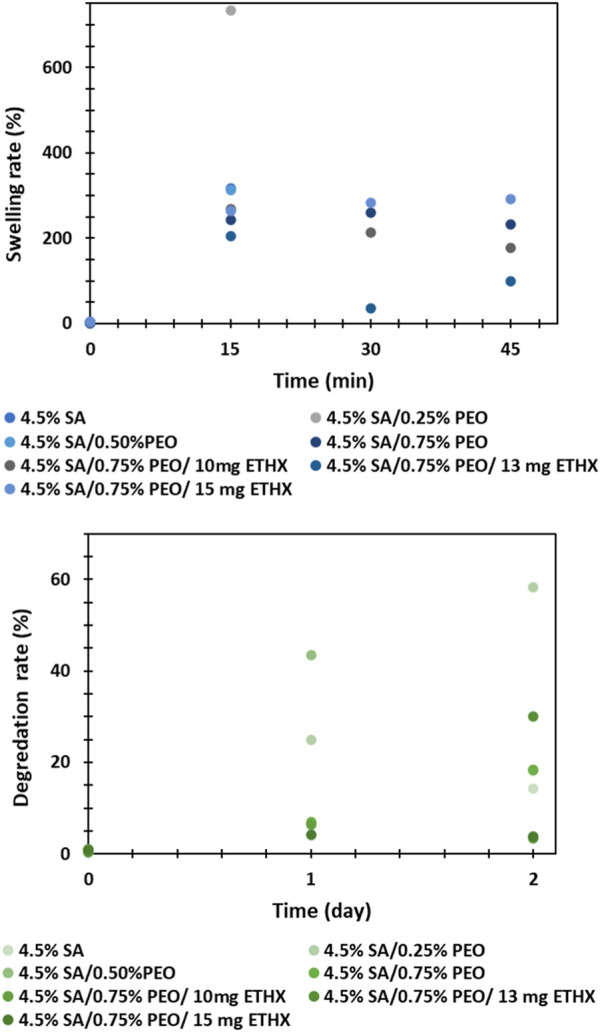
Swelling and degradation rate of the printed scaffolds within several time intervals.

The swelling and degradation rate analysis were performed *in vitro* conditions and results can be seen in the Figure 7. Even though the high swelling rate of the printed scaffolds is very advantageous for the application of polymeric scaffolds in tissue engineering and can enhance cell adhesion, the swelling and degradation capacity of the polymeric composition matrix should be controlled to provide controlled and long-lasting drug release kinetics (Chellat et al., 2000; Liu et al., 2023). In this study, it was not possible to observe swelling and degradation rate of the printed scaffolds within different time intervals due to the structure and high degradability of sodium alginate based printed scaffolds. After immersing the printed scaffolds in PBS medium, the structure of the scaffolds was easily damaged and separated into many small pieces, even though the test was repeated more than twice. For that reason, the weigh of the printed scaffolds at different times was not measured. Additionally, the effect of PEO polymer or ETHX drug in the printed scaffolds could not be compared due to a lack of data.

**FIGURE 8:**
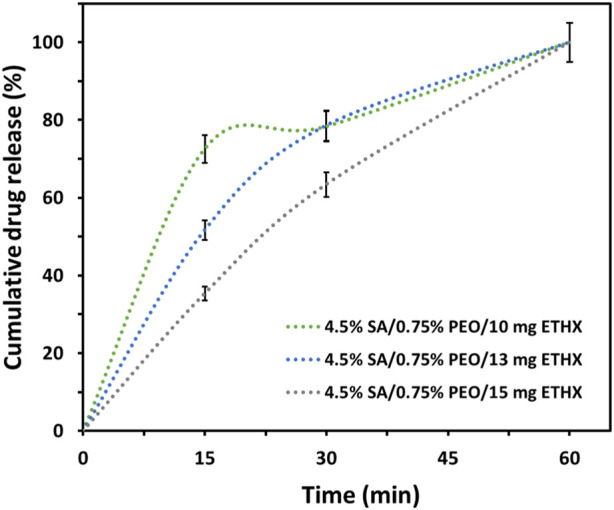
Graph of cumulative drug release kinetics of the ETHX from the printed scaffolds *in vitro* conditions (N = 2).

The drug release from each scaffold was examined in PBS (pH: 7.4). The absorbance value of the ETHX was detected at 200 nm and the cumulative drug release kinetics of the ETHX from each drug-loaded scaffold is shown in Figure 8. According to the cumulative drug release kinetics results, the drug within the scaffolds was released in different amounts and over different time periods. It can be seen that, nearly 73% of ETHX from 4.5% SA/0.75% PEO/10 mg ETHX scaffold were released in 15 min whereas approximately 52% and 36% of drug were released from 4.5% SA/0.75% PEO/13 mg ETHX and 4.5% SA/0.75% PEO/15 mg ETHX. This burst release effect in the first 15 min might be due not only to the presence of the drug on the surface of the scaffolds but also because of the high degradation rate of the SA. However, after the 30 min, controlled and slow release of the ETHX was observed. Surprisingly, the same amount of the drug from 4.5% SA/0.75% PEO/10 mg and 4.5% SA/0.75% PEO/13 mg ETHX scaffolds was released, and the release percentage was 80% at 30 min. Still, 4.5% SA/0.75% PEO/15 mg of ETHX scaffold resulted in the slowest drug release, which was 63% in 30 min. Moreover, interestingly, the drug release reached 100% in 1 h, which might be due to the high degradation and water-swelling behaviour of the SA and PEO polymers. However, further studies should be conducted based on the results of the drug release kinetics which showed that this study should be improved in terms of providing controlled and long-lasting drug release of ETHX for the treatment of epileptic seizures in the long term, and crosslinking with 3% CaCl_2_ was not enough to increase the stability of the scaffolds in PBS even though crosslinking with 3% CaCl_2_ provides a tighter junction within SA-based scaffolds (Das et al., 2008).

**FIGURE 9:**
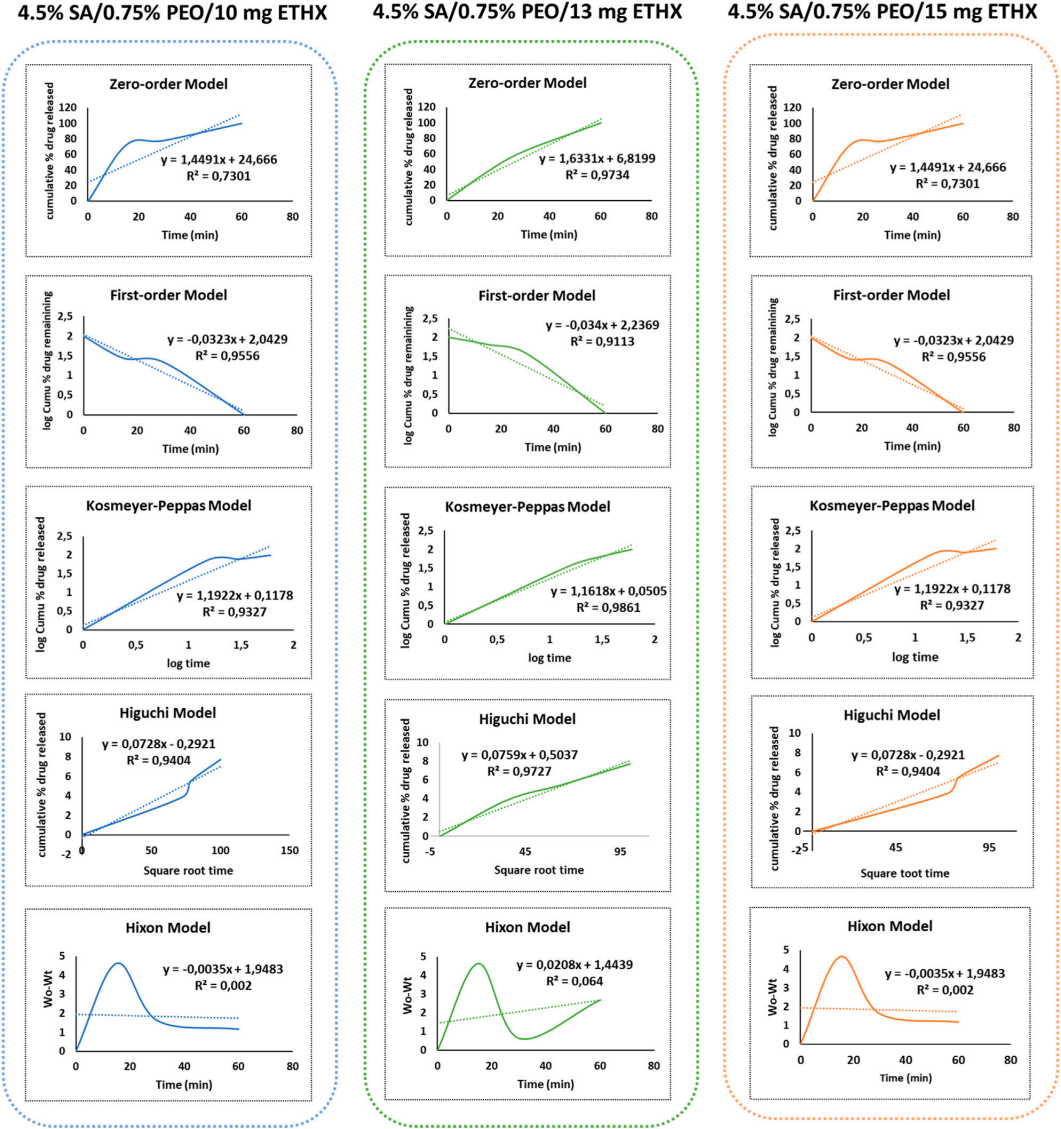
Graph of different drug release kinetic models of ETHX from the printed scaffolds *in vitro* conditions.

Additionally, different drug release kinetics were separately investigated to understand the release kinetics of released ETHX from the printed scaffolds. The release kinetics of ETHX with the Zero-Order, First-Order, Korsmeyer-Peppas, Higuchi, and Hixon-Crowell models are shown in Figure 9. In order to assess the most suitable mathematical model for the release kinetics of ETHX, the highest correlation coefficient (*R* ^2^) value should be considered, and it is possible to conclude that the first-order kinetics model, the Kosmeyer-Peppas kinetics model, and the Higuchi kinetics model resulted in more applicable behavior to optimize the drug release of ETHX from the printed scaffolds.

**FIGURE 10:**
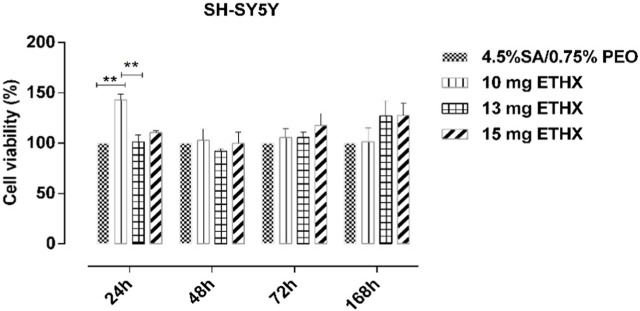
The results of the metabolic activity (relative measurement of cytotoxicity) test of SH-SY5Y cells using the MTT assay at different time intervals. N = 6. Statistical significance analysis was determined using two-way ANOVA Tukey-Kramer multiple comparisons test, with comparison to 2D., *p < 0.05, **p < 0.01, and ***p < 0.001 indicate significance levels.

**FIGURE 11:**
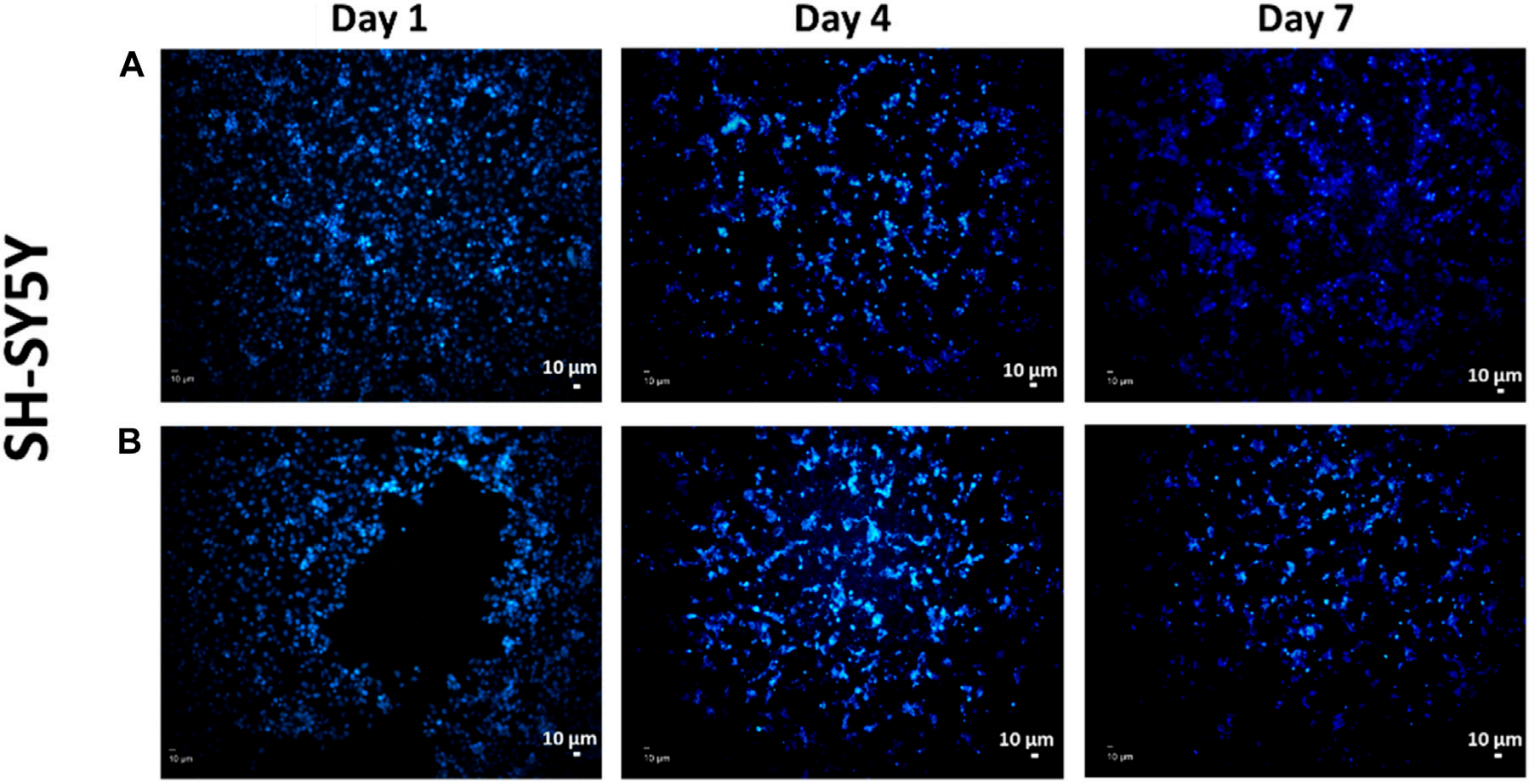
Representative DAPI-stained images of SH-SY5Y cells on the 3D-printed scaffolds of **(A)** control group 4.5% SA/0.75% PEO and **(B)** 4.5% SA/0.75% PEO/15 mg ETHX.

Since the scaffold will be in contact with the living body, it is necessary to test the cytotoxicity of the scaffolds, and it is very important to mimic the extracellular matrix for high cell adhesion on the surface of the scaffolds (Bordoni et al., 2020). Therefore, metabolic activity in terms of cytotoxicity of the scaffolds was assessed by seeding them with SH-SY5Y cells individually over several time periods. Cell metabolic activity on the scaffolds is shown in Figure 10 and representative DAPI stained images of SH-SY5Y on test samples with and without ETHX are shown in Figure 11. As control group, the alginate-based scaffold with highest PEO concentration (4.5% SA/0.75% PEO) was used. SH-SY5Y cells on the scaffolds at 24 h showed nearly more than 100% viability, confirming that SH-SY5Y cells were able to align and proliferate on the scaffolds which indicates the biocompatible environment of the scaffolds. Furthermore, SH-SY5Y cells seeded with the 4.5% SA/0.75% PEO/10 mg ETHX scaffold showed a significantly increased metabolic activity, approximately 150%, which returned to control levels at 48 h. There was no significant difference in the cells’ metabolic activity among any other scaffolds. Further studies could be conducted to fully understand the metabolic activities associated with cell viability on the printed scaffolds.

Interestingly, SH-SY5Y cells’ metabolic activity gradually increased at 72 and even 168 h, which might be because of the cells’ overcoming the adaptation problem on the scaffolds; the highest SH-SY5Y cells’ metabolic activity occurred on the scaffold of the 4.5% SA/0.75% PEO/15 mg ETHX sample. The cells metabolic activity resulted at approximately 130% on the scaffolds of 4.5% SA/0.75% PEO/13 mg ETHX and 4.5% SA/0.75% PEO/15 mg ETHX. Additionally, SH-SY5Y cells’ metabolic activity remained higher than 130% on the scaffolds of 4.5% SA/0.75% PEO/10 mg ETHX. Since SH-SY5Y cells can differentiate into nerve cells, it is crucial to provide an optimal environment for the metabolic activity of SH-SY5Y cells. Therefore, the cell metabolic activity results proved that SH-SY5Y cells were able to attach and proliferate on the scaffolds, and this study has promising potential for the treatment of neural diseases, even neural regeneration (Moon et al., 2009). Furthermore, SA and PEO composites showed the necessary qualities to mimic extracellular matrix (ECM), providing suitable conditions for SH-SY5Y cell culture. Each scaffold promoted the adhesion, proliferation, and survival of SH-SY5Y cells over several time intervals, and SH-SY5Y cells on the scaffolds might be capable of differentiating into mature neural cells (Qing et al., 2018; Innala et al., 2014).

In Figure 11, fluorescence images of the adhesion of the SH-SY5Y cells on the scaffolds over different time intervals are presented. The purpose of performing DAPI staining alone was to assess whether there were any differences in the distribution of materials and cells. Although the MTT assay was conducted to measure cell viability, DAPI staining was used to examine the spatial arrangement and localization of cells in relation to the scaffold. This allowed us to gain insights into the interactions between the material and the cells and evaluate any potential variations in cell attachment or distribution in the experimental setup. Based on the observations from the images, we can conclude that the scaffolds provided a suitable environment for the attachment and growth of SH-SY5Y cells. There seems to be a sufficient amount of cells distributed on the scaffold showing a positive cell reaction to the scaffold which indicates that the porous scaffolds provided a suitable environment for cells, so they are better adapted to it (Moukbil et al., 2020). We may infer from the findings of the cell study that the controlled release of the antiepileptic seizure drug ethosuximide from the printed scaffold had no adverse impact on the function of the cell’s metabolic activity, and in fact enhanced it considering it had been embedded in the scaffold. Therefore, this study has promising implications for the further application of treatment for epilepsy seizures.

## 4 Conclusion

The aim of this study was to develop an implantable 3D printed biodegradable scaffold enriched with ethosuximide, a proven antiepileptic agent, for the treatment of epilepsy seizures. This study not only underscores the immense potential of cutting-edge technologies like 3D printing in creating intricate and personalized medical solutions, but it also highlights the significance of integrating pharmacological agents within biodegradable matrices to enhance therapeutic efficacy. The marriage of ETHX with a biodegradable scaffold offers a promising avenue for addressing a spectrum of neurological disorders. The controlled release of ethosuximide from the scaffold provides a sustained and localized therapeutic effect, potentially minimizing adverse effects and maximizing patient comfort. Furthermore, the scaffold’s biodegradability aligns with the body’s natural processes, eliminating the need for surgical removal and reducing long-term foreign body complications. SA and PEO polymers were used at varying concentrations to create the scaffolds, with different amounts of the drug added to the composite scaffold. A control group was also established using 4.5% SA/0.75% PEO composite scaffold due to its strong mechanical properties, in which 10, 13, and 15 mg doses of ETHX were added. SEM analysis showed that the scaffolds had an ideal porosity without any blockage. The drug release kinetics displayed that the ETHX was released over a period of just 2 h, despite using a 3% CaCl_2_ crosslinking agent. *In vitro* tests on SH-SY5Y cells demonstrated that each scaffold provided an optimal environment for adhesion, proliferation, and cell survival over multiple time intervals. This study paves way for future investigations using this novel scaffold system for epilepsy.

## Data Availability

The original contributions presented in the study are included in the article/supplementary material, further inquiries can be directed to the corresponding authors.
